# Duration discrimination in the bumblebee *Bombus terrestris*

**DOI:** 10.1098/rsbl.2025.0440

**Published:** 2025-11-12

**Authors:** Alexander Davidson, Ishani Nanda, Anita Ong, Lars Chittka, Elisabetta Versace

**Affiliations:** ^1^ School of Biological and Behavioural Sciences, Queen Mary University of London, London, UK; ^2^ School of Biological Sciences, Nanyang Technological University, Singapore, Singapore

**Keywords:** duration discrimination, time perception, interval discrimination, bees, bumblebees

## Abstract

The ability to process temporal information is crucial for animal activities like foraging, mating and predator avoidance. While circadian rhythms have been extensively studied, there is limited knowledge regarding how insects process durations in the range of seconds and sub-seconds. We aimed to assess bumblebees’ (*Bombus terrestris*) ability to differentiate the durations of flashing lights in a free-foraging task. Bees were trained to associate either the long- or short-duration stimulus with a sugar reward versus an unpalatable solution until reaching a criterion and then tested without sucrose solution with the same stimuli. In experiment 1, we tested the ability to discriminate between a long stimulus (2.5 or 5 s) versus a short stimulus (0.5 or 1 s). The bees learned to discriminate between the two stimuli. To check whether bees solved the task without using the absolute difference in proximal stimulation as a cue, we ran a second experiment. In experiment 2, the flashing stimuli were presented for the same total amount of time in a cycle. Bees could discriminate between durations when the amount of stimulation in each presentation cycle was the same. This shows general learning abilities in bumblebees, that can discriminate second/sub-second intervals in visual flashing stimuli.

## 1. Introduction

Animals can use temporal cues such as the duration of time intervals to drive their choices. For instance, hummingbirds and bees schedule flower visits according to the rate at which specific flowers replenish their nectar supply [1–4]. Effective communication can also require temporal encoding and decoding of intervals, such as in the waggle dance of bees [5–7], or the mating calls of crickets, where different durations correspond to different distances and species [8]. Timescales of intervals relevant to animals vary by orders of magnitude, from years to fractions of a second. The mechanisms behind time processing in different timescales may reflect a generalized ability to keep time [9], or alternatively, there may be dedicated mechanisms for specific timescales [10]. Longer timescales such as circadian cycles depend on protein synthesis and degradation [11] that cannot account for shorter intervals in the scale of sub-seconds or seconds, which are often relevant for foraging and communication. Here, we focus on the ability of an insect, the bumblebee *Bombus terrestris*, to discriminate between visual time intervals in the scale of sub-seconds (0.5 s) to seconds (2.5–5 s) in a free-foraging task. Bees’ ability to discriminate between different timings of flashing stimuli might suggest an adaptability of the bee’s visual system to process general regularities, and their ability to learn using arbitrary temporal cues as relevant information to drive behavioural choices.

One of the first studies looking into the cognition of interval duration investigated the estimation of intervals in humans [12]. An early study with rhesus monkeys (*Macaca mulatta*) showed the ability to discriminate between two interval durations (1.5 versus 4.5 s) [13]. Pigeons can use interval timing as a cue for foraging [14]. Rats can also discriminate between durations and track the duration of intervals [15,16]. The exact neurobiological mechanisms that underlie short-duration perception in vertebrates are the object of investigation [17]. Briefly, in the pacemaker-accumulator model, durations are tracked by a regularly pulsing pacemaker neuron [18–20], whereas in the neural trajectory models, populations of neurons with specific firing rates encode the onset and offset of a duration [21]. Little is known about insects. Wasps have the ability to associate a duration (5 versus 30 min) of time with a reward [22]. Bumblebees (*Bombus impatiens*) can learn to expect a reward after a given interval (6 or 36 s) [23], although this result was challenged by work that did not find evidence of temporal control in honeybees (*Apis mellifera*) [24]. Bees are known to be good at visual learning, have keen visual acuity [25–27] and can be conditioned to reach for sucrose solution with continuous or intermittent reinforcement [28]. A preliminary study conducted on honeybees [29] suggests they are able to discriminate between flashing lights. This makes bees a good model for investigating time processing at smaller timescales using visual stimuli, though this area of research remains largely unexplored.

The present study focuses on duration discrimination of visual stimuli in the sub-second to seconds range, using a classical conditioning paradigm in free-moving bees. Bees adopt dynamic foraging strategies visiting known sources of nectar as well as uncertain sources [30–32]. They do this to gather information to optimize foraging. This feature ensures that bees sample both stimuli during training. It also means that individual behaviour is sometimes noisy, and therefore, it is essential to sample multiple individuals to assess learning performance statistically. The sub-second to seconds timescale is relevant for everyday tasks, such as navigation, foraging and communication [7,33,34]. We chose these durations based on the visual working memory of honeybees, which show a robust working memory of up to 5 s [35–38].

## 2. Methods

### (a) Subjects

**Figure 1.**
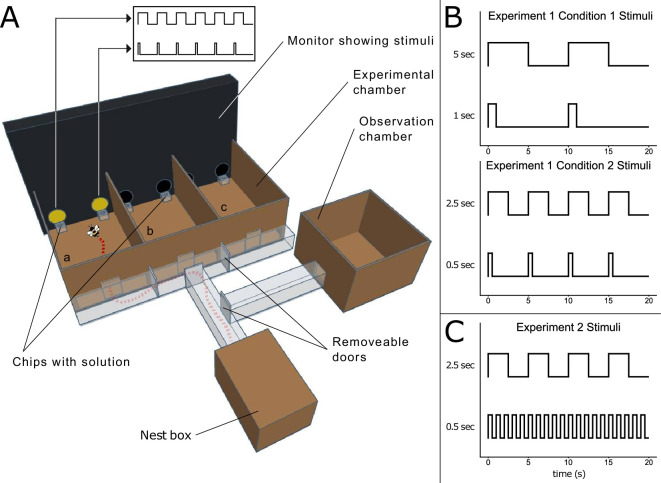
Experimental apparatus and temporal patterns of light stimuli. (A) A bee has entered the left compartment (a) of the experimental chamber. Her route is traced from the nest box. Removable doors control the available path. Before entering a compartment, the bee was locked in the tunnel directly in front for 10 s, during which time the stimuli were visible through the clear plastic and the bee was free to explore the space and observe the stimuli. A transparent, removable door blocked the entrance to the compartment. The bee would then visit compartments b and c, before returning to the nest box. A simplified representation of the stimuli is illustrated above the monitor and shown in more detail in the panels on the right. (B) The durations of stimuli in experiment 1 (5 versus 1 s in the top panel, 2.5 versus 0.5 s in the bottom panel. The peaks represent the ‘on’ state, when the stimulus was displayed. (C) The durations of stimuli in experiment 2. Over each full cycle of the long stimulus, the sum of ‘on’ states for both stimuli was equal.

We obtained bumblebees (*Bombus terrestris*) from Agralan Ltd and transferred them into a wooden nest box (figure 1). We reared bees at 22–23°C, on a 12 h light/dark cycle. Bees fed on a 20% sucrose solution available ad libitum, and we provided pollen every other day. We tested 41 bees (from 10 colonies) with a fully counterbalanced design regarding the rewarded stimulus. In experiment 1, we tested 20 bees. We trained 10 bees to associate a sugar reward with the long-duration stimulus and 10 to associate a sugar reward with the short-duration stimulus. In experiment 2, we tested 21 bees. We trained 10 bees on the long-duration stimulus and 11 on the short-duration stimulus.

### (b) Apparatus

The apparatus (figure 1A) included a hive box, an observation chamber and an experimental chamber connected by clear acrylic tunnels with removable doors to control the movement of bees. Each colony was raised in the nest box. Bees had free access to the observation chamber, where foragers were identified while visiting a 20% sucrose solution station. The observation chamber (30 × 30 × 21 cm) was covered with a clear acrylic sheet so bees could be observed feeding. A single forager per day was allowed into the experimental chamber, where bees were trained in a double-choice discrimination task until reaching a criterion and then tested.

The experimental chamber included three identical compartments (a–c in figure 1A) (25 × 17 × 10.5 cm). A white light LED strip (Govee H61902A2UK, a single SMD 5050 measured 54.4 lx at 10 cm with an Amprobe LM-120 light meter) located above the experimental chamber provided light. Acrylic chips held feeding solutions during trials. We used a computer monitor (ASUS XG258Q, 240 Hz) placed opposite to the experimental chamber to display the stimuli.

### (c) Stimuli and rewards

Stimuli were 4 cm Ø circles in yellow (hex FFFF00) on a black background. Each circle blinked on and off at the given frequency for each experiment. During the training, each stimulus could be used as a predictor of the presence of palatable and unpalatable food. Pairs of stimuli were used, shown (11.5 cm) apart so that two stimuli were present in each compartment of the experimental chamber (figure 1). Solutions were made up based on the weight of dry sugar powder or quinine hemisulfate salt and distilled water. The feeding chips were filled with 20 μl of 50% sucrose solution or 0.12% quinine solution and paired with the stimuli according to the experimental schedule.

In experiment 1, we used two conditions (figure 1B). In condition 1, we used a 10 s cycle in which the long duration was presented for 5 s and the short duration for 1 s, with a simultaneous onset. In condition 2, we used a 5 s cycle in which the long-duration stimulus was presented for 2.5 s and the short for 0.5 s with a simultaneous onset. Stimuli were presented once per cycle.

In experiment 2, we used a 5 s cycle in which the long duration stimulus flashed on and off at 2.5 s intervals, and the short-duration stimulus flashed on and off at 0.5 s intervals. This amounted to both stimuli being in the on state for 2.5 of the 5 s cycle.

### (d) Pre-training

We familiarized the bee with the experimental chamber through a three-trial pre-training procedure. For the first trial, a feeding chip with 20 μl 50% sucrose solution was placed at the entrance of each compartment to encourage the bee to enter. Two more feeding chips with sucrose solution were put in the position of the stimuli for all pre-training trials. The bee was allowed to enter the leftmost compartment first, then the middle, then the right compartment (labelled as a, b and c in figure 1A). We used plastic doors in the tunnel to restrict access to the compartments. Once the bee had entered all compartments, she was allowed back to the nest box. Once she had completed three pre-training trials, training began.

### (e) Training

Training consisted of repeated foraging bouts to the experimental chamber. We restricted the available path of the tested bee with removable doors that slotted into the tunnel (figure 1A). Before entering each compartment for a trial, the bee was locked in the tunnel facing the compartment for 10 s. This portion of the tunnel served as a transparent wall, and the entrance to the compartment was blocked by a removable transparent door. The delay ensured sufficient time to display the presentation cycle twice, allowing even the more exploratory bees the opportunity to observe the stimuli before entering the compartment. Once this time had elapsed, the bee was then allowed to enter the compartment, during which time the stimuli continued to be presented. In each compartment were two feeding chips—one with 20 μl of 50% sucrose solution and the other with 20 μl 0.12% quinine solution, placed immediately in front of the rewarded and punished stimuli, respectively. A choice was recorded as the first feeding chip she touched with any part of her body. Once the bee left the compartment, the same procedure was followed for the next two compartments. Each visit to a compartment was taken as an independent trial. The right–left stimulus position was counterbalanced between trials. Once the bee left the last compartment, she was allowed back to the nest box. We cleaned the experimental chamber with 70% ethanol solution and paper towel, removed and replaced the chips for the next trials. When the bee came back out of the nest box, the procedure was repeated. Training continued until the bee reached a set criterion of 15 correct choices in the last 20 trials. This corresponds to a *p*-value of 0.041. If a bee showed a side bias (two consecutive incorrect choices on the same side), correction trials were run, during which the rewarded stimulus was always presented on the side opposite the bias. For the first correction, a single choice on the side opposite the bias was the criterion for ending the correction. For subsequent corrections, two consecutive choices on the side opposite the bias were taken as the criterion. These correction trials were only run during the training phase. (Training data and an overview of performance during training are available in electronic supplementary material, S3 and S4.).

### (f) Test

We tested bees in 15 trials using water in both feeding chips (rewardless ‘extinction’ trials). The test procedure was the same as training except that each bout of three trials, corresponding to the visit of the three compartments, included one experimental trial in extinction and two refresher trials with sucrose and quinine (see electronic supplementary material, S1). The location of the compartment for the experimental trial with water was counterbalanced across bouts. The bee was then allowed to leave the compartment by removing the plastic door. After the three trials, the bee was made to return to the hive by restricting her path with plastic doors. Between bouts, we replaced the feeding chips and cleaned the compartments with 70% ethanol solution.

### (g) Data analysis

To assess the discrimination between the stimuli for each experiment, we analysed the population performance of extinction trials (the 15 non-reinforced trials with only water). For each individual, we calculated the overall performance as (number of correct choices)/(total number of choices) × 100.

For the population analysis, we used a generalized linear mixed model (GLMM) with a binomial distribution and logit link function for success/failure in the choice of the stimulus. We used the conditioned stimulus (short or long interval) as a fixed effect and the bee label as a random effect. To check for significant deviations from chance level, we used the intercept of the model. We tested for the significance of the stimulus using a step-wise model selection and a likelihood ratio test. We also ran an exploratory analysis to check whether the distance of the chambers from the nest box influenced the performance. We ran a linear mixed model with a normal distribution, using compartment as a fixed effect and individual bees as a random intercept to check differences in average performance by compartment. See electronic supplementary material, S4, for details on training.

Analysis was done in R version 4.3.3 using the package lme4 version 1.1-37.

## 3. Results

### (a) Experiment 1

Bees were able to discriminate between the stimuli (intercept = 0.477, s.e. = 0.122, *z* = 3.915, *p* < 0.001). Adding the conditioned stimulus (short versus long) in the model as a fixed effect did not significantly improve the model fit (χ^2^(1) = 1.127, *p* = 0.288) showing no statistical difference between the ‘short’ and ‘long’ conditions. Performance in compartment c (figure 1A) was lower than in other compartments (intercept = –0.15, s.e. = 0.072, *t*(57) = −2.07, *p* = 0.043).

### (b) Experiment 2

Bees were able to discriminate between the stimuli (intercept = 0.327, s.e. = 0.114, *z* = 2.861, *p* = 0.004). Adding the conditioned stimulus (short versus long) in the model as a fixed effect did not significantly improve the model fit (χ^2^(1) = 1.235, *p* = 0.266) showing no statistical difference between the ‘short’ and ‘long’ conditions. There was no effect of compartment on performance.

## 4. Discussion

Our results show that bumblebees can learn to discriminate between visual stimuli of different durations presented as flashing lights to guide their foraging choices. We tested bees using flashing yellow circles presented for different durations, paired with appetitive and aversive feeding solutions. In our setting, the discrimination was performed between stimuli of different duration, rather than between waiting intervals as in previous studies [1,2,4]. These stimuli are not part of bees’ natural ecological environment. Therefore, bees’ ability to learn to discriminate between these artificial stimuli shows general learning abilities through the visual modality, rather than specialized foraging strategies.

**Figure 2.**
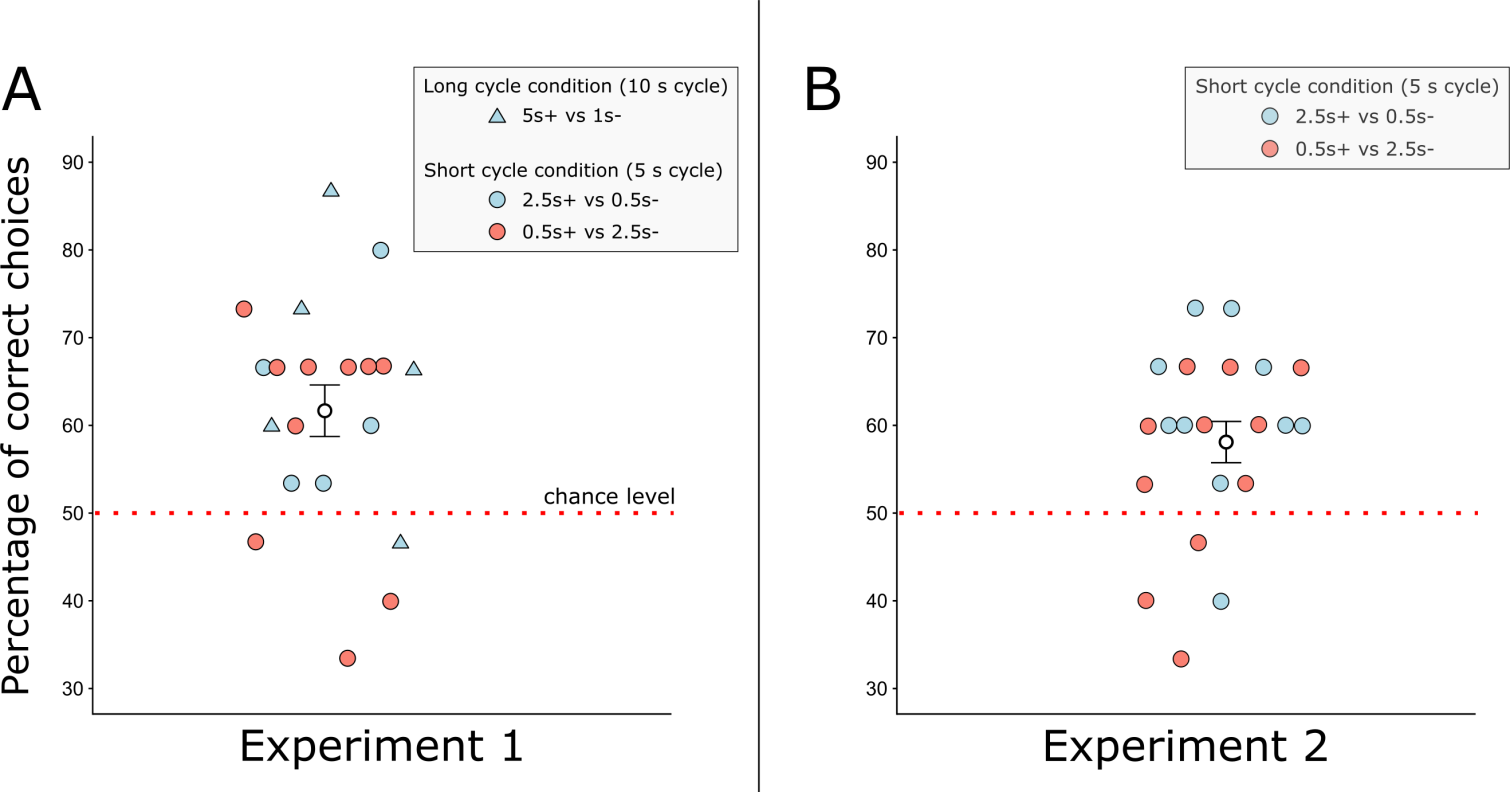
The performance of bees over 15 test trials in both experiments, where a correct choice was the choice of the stimulus that had been rewarded in training trials. Each point represents an individual bee. The dashed red line represents chance level, the white circle and error bars represent mean ± s.e. of the mean. (A) The percentage of correct choices for each bee in experiment 1, when the amount of light was not matched between conditions (correct choices/total choices × 100). (B) The percentage of correct choices for each bee in experiment 2, when the amount of light was matched between conditions (correct choices/total choices × 100).

In experiment 1, two conditions were tested: 1 versus 5 s stimuli (during a 10 s cycle) and 0.5 versus 2.5 s stimuli (during a 5 s cycle). Each flashing stimulus was presented once per cycle (same frequency in each condition). Bees learnt to discriminate between the stimuli both when the short and when the long stimulus was rewarded (figure 2A), showing that they were not driven only by spontaneous preferences and phototaxis [39,40]. Bees performed significantly worse in compartment c (figure 1A) than in a or b in experiment 1. Compartment c is furthest from the nest box and bees arrive there last. Bees’ foraging strategies are influenced by the distance from the hive [31]. However, distance is not the primary factor in guiding foraging strategies, and therefore, the effect may be small. Further experiments should explore the effect of distance versus the number of presentations. In experiment 1, the long stimulus was presented five times longer than the short stimulus. This task can be solved using either the duration of single events (on or off states of the stimuli), or using the total amount of stimulation experienced. To rule out that bees used the total amount of stimulation, we ran a second experiment in which the total amount of stimulation was matched, by having stimuli flash on and off throughout the cycle.

In experiment 2, we used 0.5 versus 2.5 s stimuli, as in experiment 1, but the total amount of light per cycle was matched between stimuli (2.5 s of light for both stimuli, figure 1C). Bees learnt to discriminate between the stimuli (figure 2B), showing that they were either processing the duration of single events (on–off states of the stimuli), or using the frequency of events in the time window of the cycle. As a frequency-based strategy was not used in experiment 1, this strongly suggests that bees used the duration of events in both experiments. We analysed group performance in learning to ask whether the behaviour of the group as a whole changes as a result of experience, since individual bees alter their foraging choices based on recent experience of reward, particularly in non-social contexts [41–43]. Bees can switch to a different flower if a previously rewarded flower is experienced as unrewarding [30,31,44,45]. Nonetheless, we found that 16/20 bees in experiment 1 and 17/21 bees in experiment 2 performed above 50% (hence, choosing the correct stimulus more frequently), although only two had statistically significant preferences at the individual level. Previous research has shown that bumblebees (*Bombus impatiens*) can learn to expect a reward, measured by extension of the proboscis, after a given interval of 6, 12 or 36 s [23]. Wasps can discriminate between durations of 5 and 30 min [22]. These studies demonstrate an ability to track the interval between or before rewards. Other work has shown that bees are able to optimize foraging bouts by learning the rates at which nectar is replenished in flowers [1,2,4]. The present study shows that bees are also able to use time cues inherent to the predictor of reward (the conditioned stimulus itself).

The ability of insects to process interval duration originating from outside the organism has been studied in naturalistic contexts, such as cricket calls [8]. While crickets have evolved to recognize calls for recognition of conspecifics and reproduction, there is no obvious ecological basis for interval discrimination of visual stimuli in bees. This points to a general learning ability in bees that can extend temporal processing to novel visual situations. It has been suggested that temporal computations might be an inherent property of neural circuits [46,47]. Whether or not general neural dynamics underlie our results, bees’ ability to discriminate between interval durations in non-naturalistic stimuli points to domain-general skills.

Temporal cognition abilities might be linked to spatial skills. For instance, during flight, bees use the rate of visual stimulation to measure speed and height [48]. Visual stimuli that intermittently appear in time might have similarities with the stimuli that flow across space during motion. A connection between the neural encoding of time and space has already been identified in vertebrate species [49,50]. Future studies should explore the underlying basis of time encoding and its connection to space processing in insects.

Overall, our findings show that bumblebees can use visual processing to discriminate between durations of external stimuli, independent of reward rates. This suggests that temporal cognition in bees extends beyond foraging strategies and relies on domain-general mechanisms. The ability to track temporal variables like duration and frequency in a non-naturalistic setting highlights a level of cognitive flexibility that warrants further investigation at the behavioural, computational and neural levels. Future research should explore whether similar mechanisms underpin time and space encoding in insects, as observed in vertebrates, and how these computations contribute to adaptive behaviour across ecological and artificial contexts. By establishing robust methods for testing duration discrimination in bees, this study opens new avenues for understanding the fundamental principles of time perception in invertebrates.

## Data Availability

All data are available in the main text or the electronic supplementary material. Supplementary material is available online [51].
